# Pulmonary function estimation using smartphone audio and deep learning

**DOI:** 10.36416/1806-3756/e20250003

**Published:** 2025-09-22

**Authors:** Gustavo de Souza dos Reis, José Baddini-Martinez, Bruno Sanches Masiero

**Affiliations:** 1. Faculdade de Engenharia Elétrica e de Computação, Universidade Estadual de Campinas, Campinas, Brasil; 2. Divisão de Pneumologia. Departamento de Medicina. Escola Paulista de Medicina. Universidade Federal de São Paulo - UNIFESP - São Paulo (SP) Brasil.

## TO THE EDITOR:

Respiratory diseases are on the rise globally, and COPD now ranks as the fourth leading cause of death worldwide. In 2021 alone, COPD claimed approximately 3.5 million lives, and 90% of deaths were in people under 70 years of age living in low- and middle-income countries.

While spirometry remains the gold standard for diagnosing COPD and monitoring pulmonary diseases, conventional spirometers cost over $2,000, limiting their availability in resource-constrained settings. Even portable alternatives may be too expensive for routine use. Mobile phones offer a promising solution to this accessibility challenge. With their widespread availability, they present an opportunity to implement cost-effective spirometry using the phone’s embedded microphone. Previous research has demonstrated the feasibility of measuring pulmonary function through breath sounds,^1^ ^-^ ^3^ although many approaches required additional equipment such as external microphones or instrumented blowpipes.^4^ ^-^ ^6^

Our approach advances this concept by analyzing the sound of a patient’s forced breathing without any external equipment, making it more accessible to health care professionals and patients alike. This shift toward equipment-free measurements opens opportunities for applying advanced analytical techniques, particularly neural networks. These computational models excel at pattern recognition, making them ideal for analyzing complex audio signals from breathing.^7^ ^,^ ^8^

It’s important to note that spirometry and audio recordings measure fundamentally different phenomena. Traditional spirometry directly measures airflow and volume, while our approach analyzes acoustic signals indirectly related to airflow. These acoustic patterns are influenced by airway anatomy, ambient acoustics, and microphone characteristics. Our objective is to establish whether these distinct techniques can derive comparable functional values, creating a reliable mapping between acoustic patterns of forced expiration and corresponding pulmonary function metrics.

**Figure 1:**
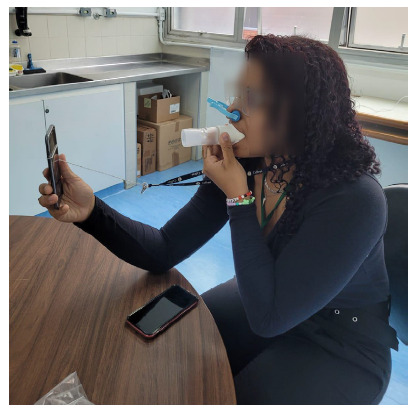
Figure diagram illustrating the standardized positioning used for smartphone spirometry. The participant performs a forced expiratory maneuver while holding a smartphone approximately 30 cm away, with the screen facing him/her, similar to taking a selfie. A nose clip and a mouth tube are used to ensure proper technique and optimize signal quality.

The authors obtained ethical approval from two Brazilian universities: *Universidade Estadual de Campinas* and *Universidade Federal de São Paulo* research ethics committees (CAAE 65695422.4.0000.5404). We collected recordings from consenting patients undergoing routine spirometry in the Pulmonary Function Laboratory of the Pulmonology Division at the *Escola Paulista de Medicina*/*Universidade Federal de São Paulo*. For each participant, a single post-bronchodilator spirometry reading was performed using conventional equipment, providing reference values for FVC, FEV_1_, and PEF. Immediately after the standard spirometry procedure, each participant performed a single forced expiration maneuver under a standardized positioning protocol. Volunteers held a Samsung Galaxy J500M/DS (Samsung Electronics; Suwon, South Korea) smartphone upright with the screen facing them at approximately 30 cm, directing their expiratory flow toward the center of the screen. To optimize signal quality and ensure reproducibility, a nose clip was applied, and a tube was placed in the mouth, as shown in Figure 1. A certified respiratory technician supervised all maneuvers to ensure proper technique, and recordings were made using the free app Audio Recorder (Samsung Electronics).

This one-to-one approach allowed for direct comparison between the clinically measured spirometry values and the audio-derived estimates for each patient. The analysis covered three key spirometry parameters: FVC, FEV_1_, and PEF. In total, we gathered 25 recordings: 7 from healthy patients, 14 from patients with obstructive diseases, and 4 from patients with restrictive disorders. The study cohort consisted of 9 males and 16 females with a mean age of 58.8 ± 13.6 years. All data was anonymized for further processing.

Audio samples were processed using the Torch Audio library, standardized to mono-channel at 48 kHz, and adjusted to a uniform duration of five seconds. To address the limited dataset size, we implemented a comprehensive three-stage data augmentation pipeline to improve model generalization and robustness. In the first stage, we applied Additive White Gaussian Noise with a controlled signal-to-noise ratio between 0 and 0.3, simulating various real-world recording conditions. The second augmentation stage involved random gain adjustment, multiplying the audio signal by a random factor between 0 and 60, helping the model become invariant to volume differences. For the third stage, we transformed each augmented audio sample into a set of three mel spectrograms with different time-frequency resolutions (window sizes: 512, 1,024, and 2,048 samples), all with a 25% frame overlap and 64 mel frequency bins. These three spectrograms were combined as channels in a single image, providing a rich multi-resolution input to the convolutional neural networks. Finally, we applied SpecAugment techniques to the spectrograms, randomly masking frequency bands and time segments to enhance model generalization.^9^ This augmentation strategy dynamically expanded the dataset, generating endless training examples from the original 25 recordings during model training.

Two convolutional neural network models were compared as regressors. The baseline architecture consisted of four consecutive blocks, each with a feature detection layer, a Rectified Linear Unit (known as ReLU) activation function, and a normalization layer. We also utilized the more advanced residual network (know as ResNet) architecture with 152 layers (ResNet152), which was pre-trained on the ImageNet database containing over 14 million images across 20,000 categories, providing a robust foundation for transfer learning to our specific task. Transfer learning was applied to the ResNet152 using three strategies: (1) Freezing-all layers were kept fixed, except the final classification layer; (2) Unfreezing-all layers were fine-tuned; and (3) Partial freezing-only the last 50 layers were fine-tuned, preserving the general feature extraction capabilities of the earlier layers while allowing the deeper layers to adapt to the specific application.

The performance of our deep learning models is quantified using the root mean squared error (RMSE) reported in Table 1. For FVC, the best-performing model was the ResNet152 with the freezing strategy, yielding an RMSE of 0.66 ± 0.27 L. For FEV_1_ and PEF, the RMSE values are approximately 0.5 L and 1.32 L/min, respectively. When compared to the average clinical values (FVC = 2.92 ± 0.89 L, FEV_1_ = 2.02 ± 0.63 L, PEF = 5.88 ± 1.94 L/min), they represent rough deviations of 28% for FVC, 35% for FEV_1_, and 20% for PEF. Although these deviations are larger than those typically seen in conventional spirometry, it is important to note that our method employs only the built-in microphone of a smartphone-without any additional hardware-to capture respiratory sounds under real-world conditions. In contrast, many existing smartphone-based or low-cost spirometry tools rely on external devices to achieve lower prediction errors. Our approach prioritizes accessibility and cost-effectiveness, making it particularly suitable for resource-limited settings.

**Table 1:** RMSE results of the tested architectures and fine-tuning techniques. RMSE measures the average magnitude of prediction errors compared to actual spirometry values. Lower values indicate better performance.

Network		RMSE		
Architecture	Fine-tuning Strategies	FVC (L)	FEV_1_ (L)	PEF (L/min)
Classic CNN	-	0.82 ± 0.15	0.49 ± 0.17	1.25 ± 0.22
ResNet152	Unfreezing	0.78 ± 0.17	0.48 ± 0.28	1.56 ± 0.51
	Partial Freezing	0.74 ± 0.23	0.52 ± 0.19	1.46 ± 0.69
	Freezing	0.66 ± 0.27	0.50 ± 0.23	1.32 ± 0.37

RMSE: root mean squared error; CNN: convolutional neural network; and ResNet152: residual network architecture with 152 layers.

In conclusion, this study demonstrates the potential of using smartphone microphones as a cost-effective and accessible alternative to traditional spirometry equipment, with deep learning models showing a promising correlation between forced expiration audio and pulmonary function parameters. While the current error margins (20-35%) are higher than clinical standards for conventional spirometry, this approach represents a significant step toward more accessible respiratory assessment tools, especially in resource-limited settings where conventional spirometers are scarce. By leveraging widely available technology and advanced machine learning techniques, we hope to contribute to more accessible respiratory health care screening worldwide.

We acknowledge the fact that the small dataset (25 samples) limits generalizability, reflecting the study’s exploratory nature. Future work should expand the dataset, refine regression models, and test advanced techniques such as recurrent neural networks or transformers to better capture temporal audio patterns. We also recognize that the current implementation does not meet established clinical pulmonary function testing accuracy standards, which typically require error margins below 5-10%. Future research will focus on reducing prediction errors to approach clinically acceptable levels for diagnostic use. Using a single device model is another limitation, as its outdated hardware may not reflect current technology. Yet, this choice serves as a “worst-case scenario,” showing that even older devices can provide valuable data. Results will likely improve with newer models featuring better microphones, and future research should include various smartphones to enhance generalizability and develop calibration protocols to manage hardware differences.
